# Mutant Cbl proteins as oncogenic drivers in myeloproliferative disorders

**DOI:** 10.18632/oncotarget.233

**Published:** 2011-03-20

**Authors:** Mayumi Naramura, Scott Nadeau, Bhopal Mohapatra, Gulzar Ahmad, Chandrani Mukhopadhyay, Martin Sattler, Srikumar M Raja, Amarnath Natarajan, Vimla Band, Hamid Band

**Affiliations:** ^1^ Eppley Institute for Research in Cancer and Allied Diseases, University of Nebraska Medical Center, Omaha, NE; ^2^ Department of Genetics, Cell Biology and Anatomy, College of Medicine, University of Nebraska Medical Center, Omaha, NE; ^3^ Department of Biochemistry & Molecular Biology, College of Medicine, University of Nebraska Medical Center, Omaha, NE; ^4^ Department of Pharmaceutical Sciences, College of Pharmacy, University of Nebraska Medical Center, Omaha, NE; ^5^ Dana Farber Cancer Institute, Harvard Medical School, Boston, MA

**Keywords:** Cbl, E3 ubiquitin ligase, leukemia, protein tyrosine kinase, signal transduction

## Abstract

Casitas B-lineage lymphoma (Cbl) family proteins are evolutionarily-conserved attenuators of protein tyrosine kinase (PTK) signaling. Biochemical analyses over the past two decades have firmly established that the negative regulatory functions of Cbl proteins are mediated through their ability to facilitate ubiquitination and thus promote degradation of PTKs. As aberrant activation of PTKs is frequently associated with oncogenesis, it has long been postulated that loss of normal Cbl functions may lead to unregulated activation of PTKs and cellular transformation. In the last few years, mutations in the *CBL* gene have been identified in a subset of human patients with myeloid malignancies. Here we discuss insights gained from the analyses of Cbl mutants both in human patients and in animal models and propose potential mechanisms of oncogenesis through this pathway.

## INTRODUCTION

While hematological malignancies represent less than 10 % of all cancer cases in the United States [1], studies into their pathogenesis have led to critical insights into the molecular mechanisms of cancer initiation and progression including fundamental paradigm shifts such as the stem cell hypothesis of cancer [2]. Hematological malignancies have also been at the forefront of studies that led to the development of molecularly targeted therapeutics [3]. Thus, identification of a novel driver of oncogenesis in hematological neoplasms is likely to shed new light on mechanisms of oncogenesis relevant to diverse types of cancer. It is in this context that recent identification of mutations in *CBL* in a small but significant proportion of patients with myeloid malignancies provides an important milestone. In this article, we review the basic functions of Cbl family proteins, survey their mutations in human patients and animal models that manifest as myeloproliferative/myelodysplastic syndromes and propose potential mechanisms of oncogenesis and possible strategies to treat patients with *CBL* mutations.

## CBL FAMILY PROTEINS

Members of the Casitas B-lineage lymphoma (Cbl) protein family are evolutionarily-conserved multi-domain regulators of signal transduction (reviewed in [4-6]). In mammals, this family includes Cbl (also known as c-Cbl), Cbl-b and Cbl-c (also known as Cbl-3 or Cbl-SL [7]). Extensive biochemical studies have demonstrated that they act primarily as attenuators of cellular signals by functioning as E3 ubiquitin ligases directed towards protein tyrosine kinase (PTK) pathways [8-10]. The N-terminal regions of all Cbl family members are highly conserved; these include the tyrosine kinase binding (TKB) domain, the RING finger (RF) domain and the short linker region between these two domains. Structural analyses have shown that the TKB domain is composed of a four-helical bundle, a calcium-binding EF hand and a variant SH2 domain [11]; together, these domains constitute a relatively unique platform that mediates specific binding to cognate phosphotyrosine-containing motifs almost exclusively found in activated PTKs. The RING finger domain and the linker region together mediates binding to E2 ubiquitin-conjugating enzymes and both of these motifs are essential for the E3 ubiquitin ligase activity of Cbl proteins [12].

The carboxyl regions of Cbl family members are more divergent; while Cbl-c possesses a relatively short region carboxyl to the RF domain, Cbl and Cbl-b contain multiple protein-protein interactions motifs including: proline-rich regions that bind to SH3 domains of a number of signaling proteins such as Src family kinases and the Grb2 adaptor protein; tyrosine residues that become phosphorylated upon cellular activation and interact with key signaling mediators such as the Vav family guanine nucleotide exchange factors, the p85 regulatory subunit of phosphatidylinositol 3-kinase (PI3K) and Crk family adaptors; and leucine zipper/ubiquitin-associated (LZ/UBA) domain that is proposed to be involved in homo- and hetero-dimerization of Cbl proteins.

Conclusions based on biochemical evidence that Cbl proteins function as attenuators of mammalian PTKs have been validated by animal models that demonstrated enhanced biological responses when Cbl family members are genetically ablated. For example, *Cbl*-null mice show increased cellularity in the hematopoietic organs [13-15] whereas *Cblb*-null mice exhibit hyper-responsiveness to immunological insults leading to autoimmunity [16, 17]; this phenotype was further augmented to a fulminant inflammatory disease when Cbl and Cbl-b were concurrently deleted in the T cell compartment [18]. Given that Cbl proteins associate with a variety of growth factor receptors, their negative regulatory roles towards PTKs engendered an expectation that Cbl proteins may function as tumor suppressors and that their mutations and/or deletions could contribute as driving or accessory oncogenic mechanisms. This hypothesis was further supported by the historical background that Cbl was originally identified as a cellular homolog of a murine viral oncogene (v-Cbl); this fibroblast-transforming gene was a fusion of the Cbl TKB domain with the viral *gag* sequences [19]. Combined with studies on the Cbl linker/RF domain mutants including a pre-B cell line-derived 70Z Cbl as well as various engineered mutants [20], these data altogether indicated that the linker/RF domains were critical for a potential tumor suppressor role of Cbl proteins. Yet, a direct role of Cbl proteins in human cancers remained elusive until recently.

**Figure:**
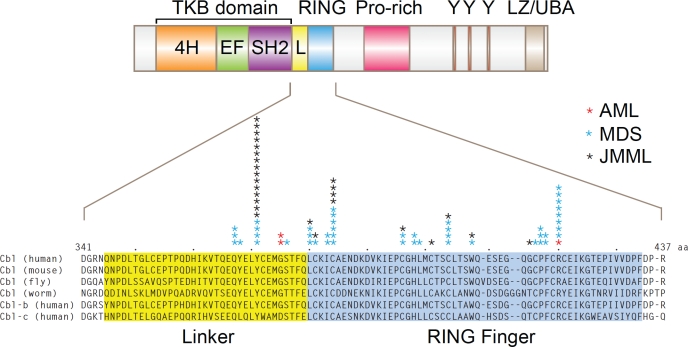
Schematic representation of mammalian Cbl, alignments of amino acid sequences for various Cbl family proteins, and positions of clinically-identified *CBL* missense mutations Mutations were compiled from published papers. TKB, tyrosine kinase-binding domain; 4H, four-helix bundle; EF, EF hand; SH2, Src-homology domain 2; L, linker; RING, “really interesting new gene” finger domain; Y, tyrosine residue; LZ/UBA, leucine zipper/ubiquitin associated domain.

## MUTANT CBL AND MYELOID MALIGNANCIES

In 2007, two groups simultaneously identified *CBL* mutations in acute myeloid leukemia (AML) patient samples [21, 22]. Since then, a number of independent studies confirmed and extended these observations and a consensus has gradually emerged on the nature of *CBL* mutations and their clinical manifestations [23-32].

First, *CBL* mutations are most frequently observed in a distinct group of myeloid disorders, namely myelodysplastic syndromes-myeloproliferative neoplasms (MDS/MPN); this subgroup of hematological malignancies includes the chronic myelomonocytic leukemia (CMML), atypical chronic myeloid leukemia (aCML) and juvenile myelomonocytic leukemia (JMML). Notably, these malignancies are often associated with hallmark genetic aberrancies that culminate in activation of the Ras-MAPK signaling pathway [33, 34]. For example, the activating mutations of *PTPN11, NRAS* and *KRAS,* and loss of Ras GTPase-activating protein gene *NF1* together account for approximately 75% cases of JMML. *CBL* mutations are now known to account for roughly half of the remaining cases. Among CMML patients, *RAS* mutations are reported in 20 to 60 % of the cases.

Second, most *CBL* mutations are missense mutations or small deletions around the linker region and within the RF domain. Where tested, these mutations have been shown to abrogate the E3 ubiquitin ligase activity of Cbl [21, 26, 28]. Complete *CBL* gene deletion, truncation, or mutations outside of the linker/RING finger regions are rare. These characteristics strongly suggest that the expression of mutant Cbl proteins confers growth and/or survival advantages over cells expressing wild-type Cbl or even those that have lost Cbl expression.

Third, a remarkable feature of patients with Cbl mutations is that the wild-type *CBL* allele is frequently lost in leukemic clones and is replaced with the mutant allele by acquired uniparental isodisomy (aUPD). Although mutations in *CBLB* and *CBLC* have also been reported, they appear to be rare compared to *CBL* mutations. In this regard, deletion of the wild-type *CBL* allele and acquisition of a second mutant allele may represent a defining oncogenic event; this hypothesis is most clearly supported by observations in JMML patients, where *CBL* mutations are often inherited as hemizygous germline mutations [32]. Apparently, a mutant Cbl protein encoded by a hemizygous *CBL* mutation is not sufficient to counter the function of Cbl family proteins encoded by the remaining wild-type *CBL* allele plus two wild-type *CBLB* alleles. This is further backed by experimental data that mutant Cbl proteins confer far more growth advantage on a *Cbl-*deficient background compared to a *CBL* wild-type background [28, 32].

Then, how do the mutant Cbl proteins function? Considering the high degree of structural similarity between Cbl and Cbl-b (and given the relatively epithelial-restricted expression of Cbl-c), it is conceivable that mutant Cbl proteins function as a dominant-negative mutant towards Cbl-b (or, as seen in some patients, towards both Cbl and Cbl-b if aUPD has not occurred) in hematopoietic cells. Phenotypic differences between mice deficient in Cbl alone or both Cbl and Cbl-b in the hematopoietic compartment are also consistent with the notion that complete loss of Cbl functions is necessary to promote myeloid malignancy. While bone marrows showed an expansion of the hematopoietic stem/progenitor compartments in both genetic backgrounds, rapidly fatal myeloproliferative disorder with peripheral organ involvement was seen only in *Cbl*, *Cblb* double-deficient mice but not in *Cbl*-null mice even at an advanced age [15, 35, 36].

Additionally, mutant Cbl proteins may function as gain-of-function oncogenes. Considering that essentially all leukemia-associated Cbl mutants possess an intact TKB and C-terminal motifs that mediate interactions with a variety of signaling proteins, it is logical to hypothesize that these E3-defective proteins will be recruited to activated PTKs (via the TKB domain) and that this will lead to the formation of a signaling complex that lacks the negative regulatory function of wild-type Cbl. In this scenario, mutant Cbl proteins will associate more persistently with signaling intermediates through phosphotyrosine-containing motifs, proline-rich regions and other potential motifs. Thus, mutant Cbl proteins are likely to serve as supramolecular scaffolds to assemble aberrant signaling complexes that can promote hyperactivation of signaling pathways normally attenuated by E3-competent wild-type Cbl. This idea is compatible with more robust transforming ability of 70Z Cbl compared to v-Cbl-equivalent sequences when expressed in fibroblasts [20] and a stronger biochemical activation of PDGFR signaling cascade by 70Z Cbl and other full-length Cbl mutants compared to v-Cbl [37].

Further support for this idea comes from analyses of a mutant Cbl knock-in mouse model developed by Langdon and colleagues in which a Cbl RING finger mutant (C379A, equivalent to C381 in human) is expressed from the endogenous *CBL* promoter [38]. While homozygous mutant mice show early lethality, heterozygous mutant mice with one wild-type *Cbl* allele do not show hematopoietic abnormalities. However, mice with one C379A mutant allele on a *Cbl*-null background (with wild-type *Cblb*) succumb to myeloid malignancies with a median survival time of 47 weeks [36]; although the disease in these mice develops later than in *Cbl*, *Cblb* double-deficient mice [35], the overall features of the disease are remarkably similar. Interestingly, Akt was constitutively activated in the C379A mutant hematopoietic cells but not in control or *Cbl-*null mutant cells [36]. This was accompanied by the enhanced phosphorylation of Y737 (corresponding to Y731 in human) of Cbl, an experimentally-proven binding site for the p85 regulatory subunit of PI3K. These findings in an *in vivo* model that phenocopies critical features of human diseases support the premise that an E3-deficient oncogenic Cbl mutant is capable of hyperactivating a key pathway through an associated signaling partner whose function would have been attenuated by wild-type Cbl. Thus, it will be highly pertinent in future studies to use this and other mouse models to identify and evaluate the activation status of PTKs that serve as physiological targets of Cbl proteins in hematopoietic stem cells, and to assess which signaling pathways are rendered hyperactive as a result of the expression of a gain-of-function Cbl mutant. Clearly, to distinguish between the dominant-negative and gain-of-function mechanisms of mutant Cbl-dependent oncogenesis, it is imperative to test their activities in cellular environments completely free of endogenous Cbl proteins. *Cbl*, *Cblb* double-deficient mice [35] as well as cells derived from these animals should be valuable research tools for this purpose.

Considering Cbl’s interaction with various signaling molecules, mutant Cbl is likely to affect a multitude of downstream pathways. We discussed the constitutive activation of the Akt-PI3K pathway above [36]. Because *CBL* mutations are seen in a sizable fraction of JMML cases, a disease entity strongly linked to hyperactivation of the Ras-Raf-MAPK pathway [34], activity of this pathway needs to be carefully evaluated. Indeed, Erk activation of LSK (Lin^-^Sca-1^+^c-Kit^+^) cells in response to Flt3 ligand stimulation is prolonged in C379A mice [36], suggesting that this is another potential pathway affected by *CBL* mutation. Molecular basis of Cbl-mediated regulation of the MAPK pathway has been proposed previously [6]. Furthermore, mutant Cbl proteins may also influence cytoskeletal reorganization and cell motility through their interaction with the Rac1 and Cdc42 pathways [39, 40]. Comprehensive structure-function analyses using ectopic expression of mutant Cbl proteins in hematopoietic stem cells and, in the long run, from its endogenous promoter through knock-in approaches should help directly test these potential mechanisms in leukemogenesis and disease progression. Finally, aberrations of other pathways such as *RUNX1, JAK2,* and *FLT3* are found in human patients with *CBL* mutations [28, 41, 42]. Therefore, modeling these additional oncogenic events in cellular and animal models should help provide a fuller picture of mutant Cbl-driven leukemogenic process.

Importantly, many of the signaling pathways that appear to be linked to mutant Cbl-driven oncogenesis are also known to be hyperactive in other cancer, and are currently being pursued actively as potential therapeutic targets. Therefore, a better understanding of the spectrum of signaling alterations provoked by mutant Cbl proteins is likely to reveal logical therapeutic strategies for patients with *CBL* mutations. Because mutant Cbl proteins may unleash pathways that are distinct from those engaged by its normal counterpart, unbiased genomic and proteomic approaches may help identify potential therapeutic targets. Finally, it may be possible to develop therapeutics that directly target mutant Cbl. Existing biochemical data indicate that mutant Cbl proteins need to interact with their PTK targets through the TKB domain to exert their transforming activity. Therefore, it is conceivable that interruption of this interaction can block mutant Cbl-driven oncogenesis. While not as widely pursued as inhibitors that target specific catalytically active sites in enzymes (such as protein tyrosine kinases), successful development of small molecule inhibitors of protein-protein interaction has begun to emerge, validating it as a practical approach [43]. The interaction interfaces that mediate the Cbl TKB domain binding to cognate phospho-peptide motifs on target PTKs have been structurally characterized and should be useful for a peptidomimetic approach to design small molecule inhibitors. This approach can be complemented with more unbiased chemical library screens. Our laboratories have established high throughput assays that are suitable for screening small molecules as well as for characterization of rationally-designed inhibitors of Cbl interaction with PTKs [44].

In conclusion, recent identification of mutations of Cbl in MDS/MPN and development of models that recapitulate features of these diseases in mice have opened exciting new avenues to translate what we have learned over the last two decades about the multi-faceted roles of Cbl family proteins as negative regulators of PTK signaling. These new findings also pose new questions about the functional roles of Cbl proteins, mechanisms by which these proteins regulate hematopoietic (and potentially other) stem cell programs, and how these new findings can be channeled into new diagnostic and therapeutic opportunities.
